# Raman spectroscopy in the study of amyloid formation and phase separation

**DOI:** 10.1042/BST20230599

**Published:** 2024-04-26

**Authors:** Sashary Ramos, Jennifer C. Lee

**Affiliations:** Laboratory of Protein Conformation and Dynamics, Biochemistry and Biophysics Center, National Heart, Lung, and Blood Institute, National Institutes of Health, Bethesda, MD, U.S.A.

**Keywords:** amyloid, imaging techniques, phase separation, protein aggregation, raman spectroscopy

## Abstract

Neurodegenerative diseases, such as Alzheimer's and Parkinson's, share a common pathological feature of amyloid structure accumulation. However, the structure-function relationship between these well-ordered, β-sheet-rich, filamentous protein deposits and disease etiology remains to be defined. Recently, an emerging hypothesis has linked phase separation, a process involved in the formation of protein condensates, to amyloid formation, suggesting that liquid protein droplets serve as loci for amyloid initiation. To elucidate how these processes contribute to disease progression, tools that can directly report on protein secondary structural changes are needed. Here, we review recent studies that have demonstrated Raman spectroscopy as a powerful vibrational technique for interrogating amyloid structures; one that offers sensitivity from the global secondary structural level to specific residues. This probe-free technique is further enhanced via coupling to a microscope, which affords structural data with spatial resolution, known as Raman spectral imaging (RSI). *In vitro* and *in cellulo* applications of RSI are discussed, highlighting studies of protein droplet aging, cellular internalization of fibrils, and Raman imaging of intracellular water. Collectively, utilization of the myriad Raman spectroscopic methods will contribute to a deeper understanding of protein conformational dynamics in the complex cellular milieu and offer potential clinical diagnostic capabilities for protein misfolding and aggregation processes in disease states.

## Introduction

Understanding the molecular intricacies of amyloid formation and phase separation is critical as these processes are connected to various neurodegenerative diseases, including Alzheimer's (AD), Parkinson's (PD), Huntington's, and amyotrophic lateral sclerosis (ALS) [1–3]. A common pathological hallmark is the abundance of amyloid aggregates, uniquely defined by their filamentous morphology and cross-β structure, in which β-strands are aligned perpendicular to the fibril axis. Phase separation is now recognized as the mechanism driving the formation of biomolecular condensates (also known as membraneless organelles) such as nucleoli, Cajal bodies, nuclear speckles, P bodies, and stress granules, where the compartmentalization of biomolecules such as nucleoproteins, DNA, and RNA perform specific biological function [4,5]. Importantly, ribonucleoproteins that participate in phase separation, such as transactive response DNA-binding protein 43 (TDP-43) and fused in sarcoma (FUS), also are enriched in protein inclusions associated with neurodegenerative diseases [6–8]. Moreover, recent findings indicate that amyloidogenic proteins such as tau and prion protein (PrP) can also phase separate into protein droplets [9,10], leading to a prevailing hypothesis suggesting that prolonged concentration of proteins in condensates could serve as a cytosolic initiation site of aggregation. To test this hypothesis, it is essential to evaluate protein conformational state(s) inside droplets and monitor how they evolve over time to define any molecular link between phase separation and amyloid formation. Here, we review contributions from the last 5 years using Raman spectroscopy and Raman spectral imaging (RSI) as emerging techniques for interrogating conformational dynamics underlying amyloidogenesis and phase separation of disease-related proteins.

## Probe-free, direct characterization of β-sheet structure in amyloids

Raman spectroscopy measures intrinsic molecular vibrations and has been demonstrated to be a robust method to obtain structural insights into amyloid fibrils, offering sensitivity from secondary structure down to the residue-level [11,12]. Raman spectroscopy is advantageous due to its ability to directly discern the development of β-sheet structure based on changes in the nature of molecular vibrations of the polypeptide backbone (amide group). This inherent label-free approach [13,14] uses a simple setup (a coherent light source, a spectrometer, and a CCD camera), relatively short collection time (seconds to minutes), and is generally non-destructive. It is compatible with different sample states (e.g. soluble and insoluble) in aqueous solution and requires minimal treatment prior to measurement.

**Figure 1.**
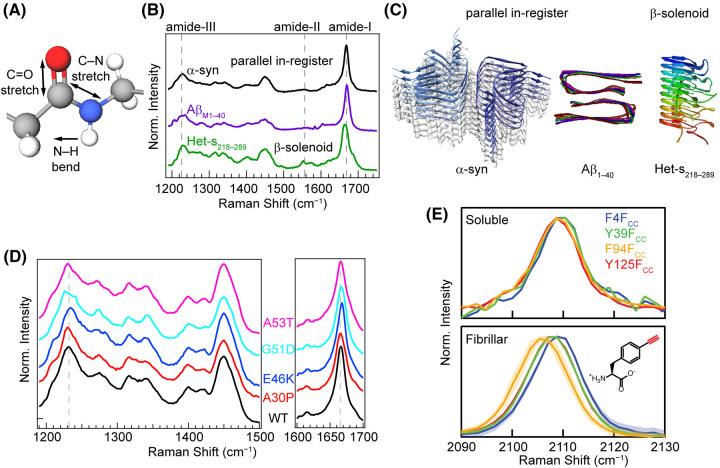
Raman spectroscopy reveals distinct structural features of amyloids. (**A**) Schematic representation of the molecular vibrations, C=O stretch, C–N stretch, and N–H bend, that contributes to the amide bands (I, II, and III) observed in the Raman spectrum. (**B**) Fingerprint region of the Raman spectra of amyloids formed by three recombinant proteins (N-acetylated α-syn, Aβ_M1–40_, and Het-s_218–289_). Spectral regions of the amide bands are indicated and dashed lines are drawn as reference guides. Data were originally published in the study by Flynn and Lee [21]. (**C**) Parallel in-register β-sheet structures composed of α-syn (left, PDB ID: 6OSJ [73]), Aβ_1–40_ (center, PDB ID: 2LMN [74]), and β-solenoid structure of the prion domain of Het-s (right, PDB ID: 2RMN [22]). (**D**) Comparison of Raman spectra from amyloids formed by PD-related α-syn mutants (A30P, E46K, G51D, and A53T) to the WT protein. Dashed lines are drawn as reference guides. Data were originally published in the study by Flynn et al. [26]. (**E**) Comparison of the C≡C stretch of F_CC_-containing α-syn mutants in the soluble (top) and fibrillar state (bottom). Chemical structure of F_CC_ is shown. Data were originally published in the study by Watson and Lee [38].

There are multiple amide bands that report on protein secondary structure characterization; the main ones are I, II, and III. For more detailed background on amide bands and analysis, we refer the reader to more extensive reviews on this topic [14–17]. Briefly, the most widely used is the amide-I band, which is primarily attributed to the carbonyl (C=O) stretch (Figure 1A). Amyloid fibrils exhibit an intense amide-I signature (∼1667–1669 cm^−1^), likely due to the ordering and long-range coupling of the β-strands in the cross-β structure. The amide-II is a weak band by comparison and most often studied by UV resonance Raman measurements [18]. The amide-III band (Figure 1A), is broad and complex; hence, is less used due to difficulty in interpretation. However, it is suggested to be more sensitive to fibril conformational differences (*vide infra*). Monitoring spectral changes in these amide bands allows for probe-free, direct identification of the secondary structure of amyloid aggregates.

### Fingerprinting different amyloids

While amyloids are generally associated with human diseases, there is a growing list of functional amyloids identified in different organisms from bacteria, fungi, and to humans [19,20]. It remains unclear as to whether there are fundamental structural differences that distinguish a disease-related amyloid from a benign or functional species. In addressing this, Raman spectroscopy was employed to investigate whether spectral differences could be observed amongst amyloids formed from recombinant proteins [21]. Raman spectra of three structurally well-defined amyloids (α-synuclein (α-syn), amyloid-β (Aβ), and Het-s_218–289_) are selected here to showcase the ‘fingerprinting’ capability of Raman spectroscopy (Figure 1B). Both pathological amyloids formed by α-syn and Aβ adopt a parallel, in-register conformation (Figure 1C), where each stacked β-strand contains the same residue, lining along the fibril axis [2]. In contrast, the functional amyloid formed by the prion-domain of the fungal protein Het-s forms a β-solenoid conformation (Figure 1C), with each molecule forming three parallel strands, wrapping up the fibril axis into a helical arrangement [22]. It is clearly evident that the amide-I signatures of the two types of cross-β structure are distinctive. Interestingly, while the amide-I bands are highly similar for α-syn and Aβ albeit with a subtle peak frequency shift, greater structural specificity is exhibited by the amide-III band, distinguishing the two pathological amyloids apart.

Work from Devitt et al. [23] has characterized not only final fibrillar structures, but used Raman spectroscopy to watch the evolution of the amide signatures from the soluble, monomeric state to the final fibrillar state. In this work, disease-related proteins with distinctive secondary structures in the monomeric state, β2-microglobulin (β-sheet) and tau (disordered), were tracked as they transformed from monomers to oligomers to fibrils. This study used principal component analysis to further deconvolute the amide-I band and mathematically show that the two proteins adopt distinctive conformations in all three states. The sensitivity of amide bands to structural perturbations coupled with multivariate data analysis techniques broaden the scope in how to view the protein aggregation process in a quantitative way.

### Sensitivity towards fibril polymorphism

An interesting feature of amyloids is the prevalence of polymorphism or different fibril structures formed by the same polypeptide chain. These structural differences can be induced by changes in the environment such as pH and ionic strength, or can be caused by post-translational modifications or disease-related mutations [24]. Polymorphism has been observed both in fibrils formed *in vitro* and those derived from patient samples [25]. Importantly, these findings suggest a structure-function relationship to disease phenotypes. While cryoelectron microscopy is groundbreaking in providing numerous near-atomic amyloid structures, it is a time-consuming and resource-intensive measurement as well as requiring extensive data processing. On the other hand, Raman spectroscopic data are collected easily and are able to discern structural differences in fibrils as reported by Flynn et al. [26]. In Figure 1D, Raman spectra of amyloid fibrils formed by PD-related mutants (A30P, E46K, G51D, and A53T) are compared with that of wild-type (WT) protein. As in the case shown in Figure 1B, the amide-III band (∼1231 cm^−1^) shows significant differences in band-shape and frequency, indicating structural changes, compared with the more subtle shifts of the amide-I bands. These structural differences were interpreted as disruption of the overall β-sheet conformation related to side-chain packing. Indeed, different structures of E46K, G51D, and A53T have since been validated with cryoEM structures, showing different interfilament interfaces [27–29].

Other polymorphic amyloids including insulin have been described by Ishigaki et al. [30]. Here, multivariate analysis was performed to determine contributions of various protein peaks in detecting polymorphism induced by different solution conditions. Furthermore, fibrils formed by phosphorylated tau, a post-translational modification associated with AD, exhibit unique Raman signatures [31]. Most recently, work by Harper et al. [32] on short peptides derived from amylin and Aβ has shown the potential of obtaining better resolved structural information by applying experimentally derived constraints from the amide-III band to molecular dynamics simulations. As this technique develops, it may be possible to move beyond ‘fingerprinting’ to structure determination. Altogether, the intrinsic sensitivity to polymorphism is promising for the development of Raman spectroscopy as a diagnostics tool for early disease detection by evaluating protein structural differences from patient samples.

### Segmental and site-specific Raman probes of amyloid structure

As detailed above, intrinsic amide vibrations can be utilized to obtain intricate secondary structural information on amyloid fibrils; however, isotopic labeling and incorporation of biorthogonal vibrations are excellent methods for extracting region/site-specific information from Raman spectroscopy. To gain information at the residue-level, one common approach in vibrational spectroscopy is through isotopic labeling, wherein specific vibrational modes such as the amide-I can be shifted for individual amino acids or protein segments. For example, it is valuable to be able to differentiate which polypeptide region first adopts β-sheet structure as the protein aggregates, which was achieved in segmentally ^13^C/^12^C-labeled α-syn [33]. This approach is also beneficial in unambiguous localization of cellularly internalized ^15^N/^13^C/^2^H-labeled α-syn fibrils as the ^13^C–^2^H stretch is found in the cellularly quiet spectral region (∼1900–2500 cm^−1^) where no other biomolecular vibrations exist [34].

Lastly, incorporation of unnatural amino acids can provide site-specific information at the individual residue-level. In particular, amino acids that contain carbon-carbon triple bonds are especially useful Raman probe, such as homopropargylglycine, a methionine analog [35], and 4-ethynyl-l-phenylalanine (F_CC_, Figure 1E, inset) owing to the fact that the alkyne stretch is environmentally sensitive [36–38]. Upon fibril formation, the F_CC_ frequency will shift if this sidechain is transferred from a polar environment to the hydrophobic surroundings of the amyloid core. This site-specific dependence comparing soluble to fibrillar states is exemplified in Figure 1E, indicating F94F_CC_ and Y39F_CC_ are proximal to the amyloid core whereas F4F_CC_ remains water-exposed. Like the ^13^C–^2^H stretch, the C≡C stretch resides in the cellularly quiet region, making it a suitable probe for cellular imaging as well (*vide infra*).

## RSI provides molecular insights with spatial context

Raman spectroscopy is a flexible tool that can be easily paired to a microscope, allowing for probe-free biomolecular imaging of cells and tissues with diffraction-limited spatial resolution [39]. Therefore, with RSI, one can obtain a specific spectrum associated with individual regions of interest in a sample. There is a wealth of information to be gained from a single Raman spectrum. Various maps can be generated for different biomolecules simultaneously (i.e. multiplexing). For example, one can distinguish nuclear vs. cytosolic regions within a cell by using frequencies unique for nucleotides and proteins, respectively. Lipid- and protein-rich regions such as lipid droplets and nucleoli can also be visualized. This measurement is translatable to other systems where the ability to obtain spatial information about biomolecular speciation is critical, such as studies on phase separation, where proteins along with biomolecules (i.e. RNA and metabolites) partition into a dilute and condensed phase.

### Probing liquid condensates with RSI

**Figure 2.**
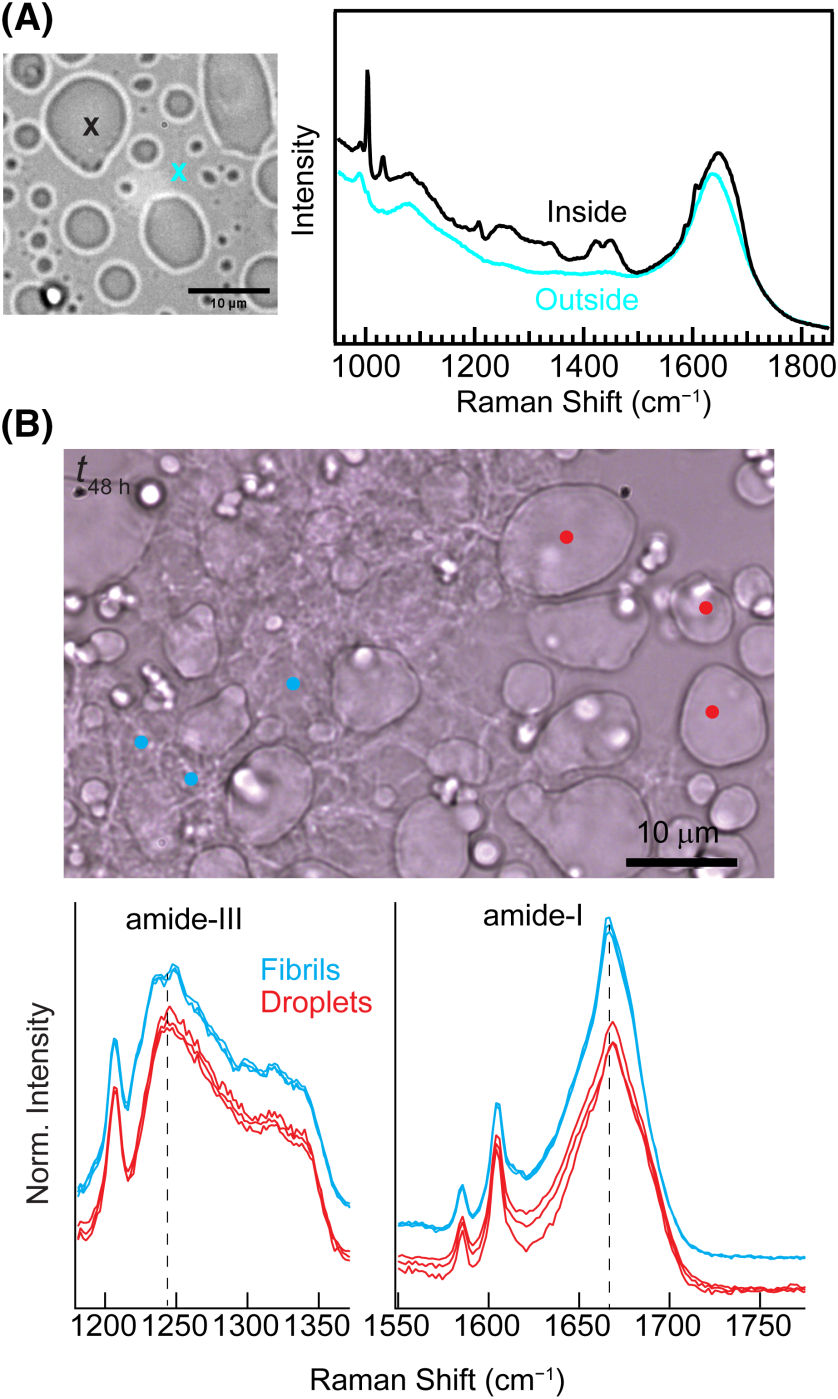
Raman spectral imaging of TDP-43_CTD_ droplets. (**A**) Bright-field image is shown on the left with **x** indicating spatial locations of Raman measurements. Unprocessed Raman spectral data collected inside (cyan) vs. outside (black) of a protein condensate are shown on the right. Protein signatures are evident inside whereas there is only bulk water outside the droplet. (**B**) Bright-field image after 48 h incubation is shown on top with circles indicating spatial locations of Raman measurements. Cyan and red indicate fibril vs. droplet morphology. Distinctive Raman spectra for the two types of aggregates in the amide-III and amide-I are shown on the bottom panels. Respective spectral regions are indicated and dashed lines are drawn as reference guides. Spectra are offset for clarity and normalized to the Phe breathing peak for comparison. Data were originally published in the study by Shuster and Lee [40].

Phase separation is a reversible process in which proteins can form condensates based on cellular needs [5]. However, as phase-separated condensates age, they can become part of the aggregation pathway, leading to amorphous or fibrillar aggregates. While the relationship between fibril formation and phase separation is not well understood, their connection is well-documented. Recent studies have leveraged RSI to elucidate this possible interplay between phase separation and amyloid formation, offering secondary structural characterization as a function of location (e.g. inside a protein droplet) and time (Figure 2). In Shuster and Lee [40], aging of the TDP-43 C-terminal domain (TDP-43_CTD_), an N-terminally truncated variant implicated in ALS and frontotemporal lobar degeneration [41], was monitored by RSI. RSI allows for a spectrum to be collected from within a protein droplet and compared with the bulk solution outside (Figure 2A). After 48 h, fibrillar aggregates appear along with droplets (Figure 2B, top). Interestingly, both droplets and fibrils contain substantial β-sheet content as assessed by their Raman spectra (Figure 2B, bottom); however, it is apparent that the amide-I band at 1669 cm^−1^ is narrower for the fibrils, indicating an increased β-sheet structure in the filamentous aggregates compared with the aged droplets. This structural difference is also observed in the amide-III band. This study exemplifies the power and capability of RSI to watch the liquid-to-solid transition of droplets; the spectroscopic differentiation of the two classes of non-filamentous and fibrillar aggregates would not have been possible without the spatial resolution of a microscope. These results are biologically relevant because they support the hypothesis that amyloid polymorphism can be a result of cellular compartmentalization (i.e. within biomolecular condensates vs. cytosolic solution).

The laboratory of Mukhopadhyay has led investigations delving into the conformational evolution of PrP within liquid-like condensates. We refer the readers to a detailed review on the role of phase separation pertaining to PrP biology [42]. Similar to TDP-43_CTD_, the disease-related PrP mutant, PrP Y145Stop, spontaneously phase separates with the addition of salt, and a higher β-sheet content was measured based upon analysis of the amide-I band [43]. Additionally, the intensity ratio of aromatic residues (Trp and Tyr) were used to determine changes associated with the hydration and structure of the protein upon phase separation and amyloid formation.

Other Raman-based methods, such as coherent anti-Stokes Raman scattering spectroscopy have been used to study phase separation. Fawzi has led investigations into the conformational evolution of FUS within liquid-like condensates, where Raman spectroscopy served as a crucial complementary technique to other biophysical methods such as fluorescence microscopy, molecular dynamics simulations, and NMR [44]. For the low-complexity domain of FUS, Murthy et al. [44] also noted narrowing of the amide-I and distinctive side-chain vibrations of Tyr residues suggesting conformational restriction upon aggregation, again highlighting the site-specific sensitivity of Raman spectroscopy.

The spatial context and multiplexing capability of RSI are not only useful for probing biomolecular vibrations, but can add to investigations of solvent contributions and hydration. The need to consider the involvement of the solvent is supported by recent terahertz spectroscopic studies, which have suggested that water molecules play a key role in phase separation [45–47]. With the added advantage of spatial context, Choi et al. [48] used RSI and model proteins to compare water populations inside and outside of protein condensates. Of note, D_2_O was used to shift the O–H stretching mode of water to lower energy to avoid spectral interference from the protein. An interplay between water and phase separation was suggested as different contributions to tetrahedral and distorted water populations were observed inside vs. outside the droplets.

### Raman mapping of cellularly internalized fibrils

**Figure 3.**
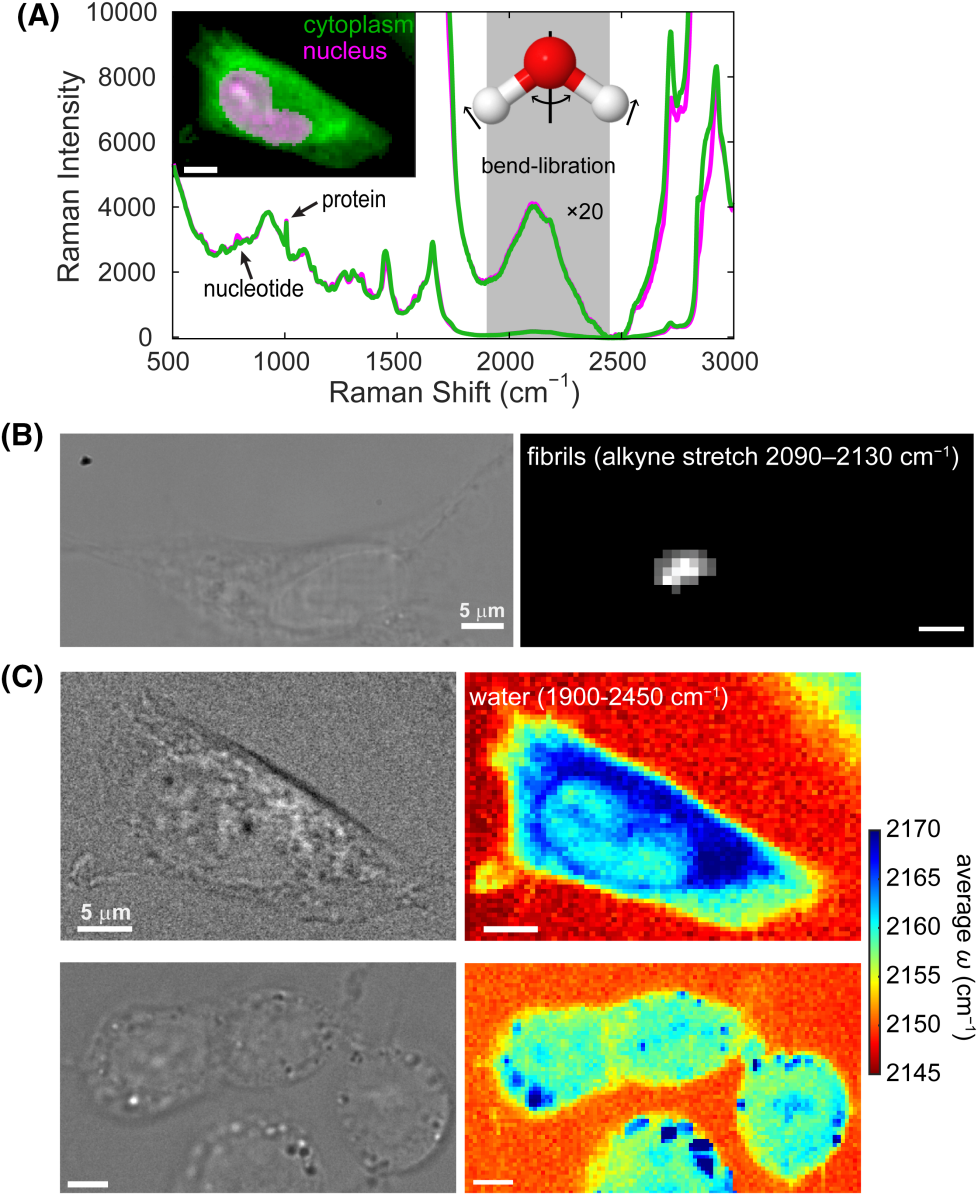
Cellular mapping by Raman spectral imaging. (**A**) Averaged Raman spectra collected from within the cytoplasm (green) and nucleus (magenta) of an SH-SY5Y cell. The inset depicts the composite image generated when taking the integrated area of the nucleotide-associated vibration (775–790 cm^−1^) and protein associated vibration of the phenylalanine ring breathing mode (995–1010 cm^−1^). (**B**) Bright-field image of an SH-SY5Y cell treated with F_CC_-labeled α-syn fibrils (left) and the localization of the internalized fibrils by the alkyne stretch (right). Data were originally published in the study by Watson and Lee [38]. (**C**) Bright-field images (left) of SH-SY5Y cells and corresponding hydration maps (right). Data for (A) and (C) were originally published in the study by Ramos and Lee [63].

Transitioning RSI into a standard tool for *in cellulo* studies can further expand our understanding of biological processes without the need for exogenous labels. For example, the cytosol and nucleus can be distinguished by using endogenous biomolecular vibrations such as the pyrimidine ring breathing mode for nucleotides and the phenylalanine ring breathing mode for proteins (Figure 3A, inset). As mentioned above, vibrations within the cellular spectral quiet region (Figure 3A, gray) such as the ^13^C–^2^H stretch [34] and unnatural amino acid, F_CC_ [38], can be used to serve as a unique label for unambiguous identification and localization of protein of interest within a cell. Shown in Figure 3B, internalized F_CC_-α-syn fibrils are clearly resolved by RSI using the alkyne peak. Beyond localization of the fibrils, RSI allows for simultaneous characterization of other biomolecules (e.g. lipids and proteins) that colocalize with fibrils and address whether there are changes in their levels. Further detailed studies can thus illuminate the cellular uptake process of exogenously added fibrils, which is pertinent as it is related to the cell-to-cell transmission of fibrils in disease progression. Indeed, several studies are interrogating amyloid structures in *ex vivo* patient tissues [49–54].

### Raman imaging of biological water

As the field moves toward *in cellulo* studies, the complexity of the cell must be addressed, such as the role of hydration in protein aggregation. Camino et al. have studied how protein hydration affects primary nucleation of α-syn [55,56] and provided a perspective on the role of water in amyloid aggregation [56]. For a broader background on biological hydration we refer to the readers to more in-depth reviews on the topic [57–59]. To understand the role of water on amyloid formation in a cellular context, it is necessary to first understand the hydration environment of a cell. The study of intracellular hydration is important because of the ubiquity of water in cellular processes; however, there are currently limited techniques available to examine the chemical nature of water in cellular environments with spatial context. Recently, RSI has been demonstrated as a viable technique to fill this gap. In particular, the intense O–H stretch of water has been employed to differentiate cytosolic and nuclear water as well as lipid-associated water [60,61]. Stimulated Raman excited fluorescence microscopy, a more involved Raman-related technique, has also shown differences between water in the nucleus vs. the cytosol indirectly by probing with an exogenous, environmentally sensitive nitrile probe, which has a resolved signal within the spectral quiet region [62].

In the latest development, a novel approach was used to directly characterize intracellular water molecules by exploiting the bend-libration combination band of water as a Raman imaging probe of hydration [63]. To the best of our knowledge, this is the first report on the use of the water bend-libration in the investigation of water structure in human cells. The bend-libration band is an environmentally sensitive, coupled vibration between the H–O–H bend [64] and librational motions [65–67], residing in the spectral quiet region (Figure 3A), free from interference from endogenous biomolecules, unlike the O–H stretch, which overlaps with C–H and N–H stretching bands. By using RSI, water hydration maps are generated based on the unique spectral features (mean frequency) of the bend-libration band (Figure 3C, right). Heterogeneity of intracellular hydration is clearly observed from the peripheral, into the cytosol and the nucleus, where subcellular compartments can be discerned. Importantly, nucleoli within the nucleus and cytosolic lipid droplets were visualized directly, a feat not previously achieved by vibrational imaging. The observed increased mean frequency within the cell compared with extracellular water is thought to be due to stronger H-bonding and/or more ordered-water environments. This superb sensitivity is due to coupling with low-frequency librations, which are responsive to greater water organization and structure [68] in these subcellular compartments (i.e. confinement) due to increases in molecular density (i.e. macromolecular crowding). Chemical insights into distinctive water populations within cellular compartments is highly relevant. These studies are foundational for future research in exploration of protein dynamics within living cells and organisms.

## Conclusions and outlook

The studies highlighted here showcase the success of Raman spectroscopy in gaining structural and mechanistic insights into amyloid fibrils and protein condensates. These studies are important in the understanding of their relationships to disease etiology. With RSI, chemical mapping within cells and tissues further demonstrates the utility and versatility of Raman spectroscopy, which has the potential to be used as a diagnostic tool in the future. It is anticipated that the work reviewed here will not only motivate experimentalists to use Raman spectroscopy as a standard technique for fibril structural characterization *in vitro* but also stimulate interest in the development of a theoretical and computational framework [69–72] to complement the ever-increasing quantity of Raman spectral data. As this research moves forward, new methods are needed to improve spectral interpretation in order to enhance the impact of this technique. Importantly, we believe that as more complex biological systems are interrogated, a better chemical understanding of intracellular hydration is crucial. We postulate that there may be potential for the design and/or manipulation of hydration states as a new therapeutic strategy as we develop a model describing how changes in hydration influence intracellular protein misfolding and aggregation.

## Perspectives

- Raman spectroscopy is an accessible, label-free approach to directly obtain protein secondary structural information from a broad array of biological samples. This technique is sensitive to amyloid fibril polymorphism and reveals mechanistic insights into the relationship between phase separation and amyloid formation.
- RSI provides spatial context with molecular information, making it a powerful tool for studies of biological samples.
- RSI can be utilized to study protein conformational dynamics and hydration in complex cellular environments, offering potential clinical diagnostic capabilities of protein misfolding and aggregation processes in disease states.

## Abbreviations

ALS: amyotrophic lateral sclerosis
α-syn: α-synuclein
FUS: fused in sarcoma
PrP: prion protein
RSI: Raman spectral imaging
TDP-43_CTD_: TDP-43 C-terminal domain
WT: wild-type
